# Central Neuropathic Pain and Profiles of Quantitative Electroencephalography in Multiple Sclerosis Patients

**DOI:** 10.3389/fneur.2019.01380

**Published:** 2020-01-21

**Authors:** Nataliya A. Krupina, Maxim V. Churyukanov, Mikhail L. Kukushkin, Nikolay N. Yakhno

**Affiliations:** ^1^Laboratory of General Pathology of the Nervous System, The Institute of General Pathology and Pathophysiology, Moscow, Russia; ^2^I.M. Sechenov First Moscow State Medical University (Sechenov University), Moscow, Russia; ^3^Clinic of Pain Study and Treatment, B.V. Petrovsky Russian Scientific Surgery Center, Moscow, Russia; ^4^Laboratory of Fundamental and Applied Problems of Pain, The Institute of General Pathology and Pathophysiology, Moscow, Russia; ^5^Scientific and Research Department of Neurology, I.M. Sechenov First Moscow State Medical University (Sechenov University), Moscow, Russia

**Keywords:** multiple sclerosis, central neuropathic pain, quantitative EEG, spectral power, peak frequency

## Abstract

Pain has a significant impact on the quality of life of patients with multiple sclerosis (MS). However, the neurophysiological mechanisms of central neuropathic pain in a MS course are not known. We hypothesized that changes in power spectral density (PSD) that take place in the electroencephalography (EEG) of MS patients with and without the central neuropathic pain (CNP) would differ. The study aimed to assess the features of quantitative EEG using the PSD indicator along with peak frequencies in the standard frequency bands in MS patients with and without CNP. We have analyzed the quantitative spectral content of the EEG at a resting state in 12 MS patients with CNP, 12 MS patients without CNP, and 12 gender- and age-matched healthy controls using fast Fourier transformation. Based on the ANOVA, at the group level, the theta band absolute and relative PSD showed an increase, whereas alpha band relative PSD showed a decrease in MS patients both with and without CNP. However, only in MS with CNP group, the absolute and relative PSD in the beta1 and beta2 bands increased and exceeded that in patients without pain. Only MS patients with CNP demonstrated the significantly increased absolute PSD for the theta, beta1, and beta2 frequency bands in most regions of interest. In the theta band, MS patients with CNP displayed the increase in absolute spectral power for the mid-temporal derivation of the right hemisphere and the increase in relative spectral power for the prefrontal derivation of this hemisphere. In the beta1 band, the increase in absolute spectral power was observed for the three temporal derivations of the right hemisphere, whereas in the beta2 band, for the occipital, parietal, and temporal lobes of both hemispheres. In the alpha band, only a relative spectral power decrease was revealed for the occipital lobes of both hemispheres and parietal lobe of the right hemisphere. In MS patients with CNP, the frequencies of the dominant spectral power (peak frequencies) in the high-frequency beta band were higher than in the healthy control in posterior areas of the left hemisphere. Data could represent central nervous system alterations related to central neuropathic pain in MS patients that lead to the disturbances in cortical communication.

## Introduction

Pain has a significant impact on the quality of life of patients with multiple sclerosis (MS) (1–6). According to various data, pain occurs in 50–85% of MS patients, of whom 30–58% suffers from central neuropathic pain (CNP) (7–10). However, so far, it is unclear whether central neuropathic pain affects the multiple sclerosis electroencephalography (EEG) patterns, and, if so, how.

The International Association for the Study of Pain defines neuropathic pain as pain originating from a lesion or disease of the somatosensory nervous system (11). There is no definite view of the genesis of CNP, including MS (12–14). The lesions of spinothalamic–cortical tracts and failure to conduct nociceptive information are believed to be determinants for CNP development (15, 16). This view was based on the results of the studies where clinical and neurophysiological methods were used (9, 17). The predisposing role of psychological and behavioral factors for CNP in MS patients is a matter of debate (18).

MS pathophysiology contains two dissimilar arms: the inflammatory demyelination and the neurodegeneration running in parallel (3). Axonal damage along with neuronal loss occurs from the beginning of disease process and leads to progressive and permanent disability. MS potentially affects human CNS at all levels. Magnetic resonance imaging studies show cortical and corpus callosum damages in MS patients (19, 20). As a result of axonal damage and widespread gray matter pathology, one can expect a deficit in cortico-cortical and cortico-subcortical connectivity leading to EEG alterations. Numerous studies point to EEG abnormalities in 20–60% (21, 22) or even in 40–79% of MS patients (23). More typical findings are an increase in slow frequencies (theta and delta) and a decrease in the alpha band especially related to cognitive dysfunctions; occasionally, paroxysmal activity, and focal slow waves or localized flattened EEG activity may be found (22–25). Different works have looked at possible relationships between EEG activity and some aspects of the MS disease. There are conflicting data on the correlation between EEG changes and the disability score in MS patients (26, 27). In recent studies assessing cognitive impairment in MS, changes in the high EEG spectrum (beta2 and gamma) localized to the anterior regions of the right hemisphere and bilaterally to the posterior areas of the scalp were revealed (28, 29). No significant correlations between quantitative EEG (QEEG) changes and the other variables analyzed, including behavioral performance, were observed. In general, EEG spectral power is considered to be of little use in MS (30).

As for CNP, EEG changes in patients with neuropathic pain are thought to occur due to the development of thalamocortical dysrhythmias (15, 31–34). EEG analysis for neuropathic pain revealed changes not only in the standard frequency ranges in general but also in some subbands. In patients with neurogenic pain, Sarnthein and Jeanmonod (35) determined the EEG power peak in the standard theta band (θ, 4–9 Hz), when the alpha peak in the standard alpha band (α, 9–13 Hz) was reduced or even not present. Vuckovic et al. (36) identified predictors of neuropathic pain in patients with spinal cord injury showing the reduction in the EEG reactivity to opening eyes in the alpha band and reduced alpha power. Slowing of the dominant peak and EEG spectral power overactivations in the theta (with maximal differences in the high theta frequencies) and beta (in the low beta frequencies, beta1 band) power localized to multiple pain-associated areas were the most apparent characteristics in patients with neurogenic pain (37, 38). Under painful stimulation, healthy volunteers demonstrated a most pronounced decrease in alpha amplitude and several changes evidenced for the increase in the high beta frequencies (18.5–24 Hz) (32). Neurofeedback training for treatment of central neuropathic pain showed the association between pain reduction and suppression of theta power in the standard band and beta2 power in the 20–30 Hz band (39). A number of EEG studies have also shown the association between neuropathic pain and beta/gamma overactivations (32–34). However, changes in the power spectral density in the gamma frequency range are often associated with the attentional processing and cognitive impairment (28, 40). In patients with severe chronic neuropathic pain, higher pain ratings correlated consistently positive with EEG power values, while larger psychopathology correlated with lower frequency values (4).

Given the above, patients with MS and patients with CNP have several similar changes in the EEG. First of all, one might draw attention to an increase in spectral power in the theta range and a decrease in the alpha activity. However, only patients with CNP demonstrated an increase in the spectral power in the high-frequency bands. The question remains how the neuropathic pain could change the EEG pattern characteristic for MS, and vice versa, that is, what is the interaction between MS and chronic neuropathic pain as judging by the EEG indices? How will thalamocortical dysrhythmia related to CNP manifest in patients with multiple sclerosis? What mechanisms can underlie the influences if they appeared? Comparative neurophysiological studies in MS with and without CNP may be a new approach to improving our knowledge of both MS and CNP genesis. We consider the present study as the first step in finding answers to questions posed.

This work aimed to identify the features of QEEG using the PSD indicator along with peak frequencies in the standard frequency bands in MS patients with and without CNP.

## Materials and Methods

### Patients and Healthy Controls

This study was carried out in accordance with the recommendations of the Ethical Committee of the Institute of General Pathology and Pathophysiology (the project approval protocol Number 5 of November 25, 2016) and was approved by the Ethical Committee (final approval protocol Number 1a of April 03, 2018). All participants signed informed consent after a complete explanation of the study in accordance with the Helsinki Declaration of 1964 with all subsequent amendments.

All the patients underwent complex assessment, which included a clinical neurological examination. Neurological impairment was estimated according to Kurtzke's Expanded Disability Status Scale (EDSS scores) (41).

Diagnosis of definite MS was based on clinical and neuroradiological data, according to the criteria of McDonald et al. (42).

Inclusion criteria for our study were clinical remission of the disease in the last 1 month before the study and the absence of corticosteroid treatment for at least 1 month. No cases of epilepsy were diagnosed in MS patients included.

The group of MS patients was divided into two clinical subgroups based on CNP presence. MS patients were classified as either having CNP or not. Most of the patients reported pain in more than one anatomical location. The most commonly reported pain areas were lower extremities and the upper extremities. Five patients reported pain on the left side of the body and five on the right side, and two patients experienced pain both on the right and on the left. Assessment took place according to the complaints of pain with descriptors for the neuropathic pain, symptoms of CNS lesion that is sensation disorders (hypoesthesia, hyperesthesia, paresthesia, or dysesthesia) in a body territory with decreased or increased sensation to touch, pinprick, warmth or cold at bedside examination, and had no history or clinical evidence of peripheral neuropathic pain. Furthermore, nociceptive musculoskeletal, spasticity-related, and visceral pain conditions had to be either excluded. Pain syndrome was assessed using the 10-point Visual Analog Scale (VAS). The mean VAS score for the group of MS patients with CNP was 5.4 ± 0.6 (mean ± SEM).

Exclusion criteria for MS patients with and without CNP were the same: other known neurological diseases (including peripheral nerve damage), cancer, renal disease, severe psychiatric disease, diabetes mellitus, and Mini-Mental State Examination score <24 (43).

Among comorbid disorders, some patients in MS groups with and without CNP had occasional tension headaches, migraines, arterial hypertension, biliary dyskinesia, irritable bowel syndrome, peptic ulcer disease, and psoriasis.

The healthy control (HC) group included volunteers with no history of neurological disorders and not allowed to suffer from any pain.

The characteristics of the groups examined are given in Table 1. All study participants were right-handed.

**Table 1:** Baseline characteristics of the groups.

**Groups** **(number of participants)**	**Males/females**	**The average age, years** **(min – max)**	**The number of patients with a clinical type of the disease course**	**The average duration of the disease, years**	**EDSS scores**
MS with CNP (*n* = 12)	3/9	36.6 ± 3.2 (23–57)	RRMS – 10 PPMS – 2	7.2 ± 1.2	2.8 [2.0; 3.0]
MS without CNP (*n* = 12)	4/8	42.9 ± 2.8 (30–55)	RRMS – 9 PPMS – 1 SPMS – 2	7.2 ± 1.8	2.5 [2.0; 3.0]
Healthy control (HC) (*n* = 12)	4/8	40.3 ± 4.0 (22–65)	–	–	–

*RRMS, relapsing-remitting multiple sclerosis; PPMS, primary progressive multiple sclerosis; SPMS, secondary progressive multiple sclerosis, according to Lublin and Reingold (44). The average age and the average duration of the disease presented as Mean ± S.E.M; EDSS scores—as Median with lower and upper quartiles*.

### EEG Recording

EEG was administered to MS patients during remission, usually after the course of corticosteroids. MS patients with CNP did not complain of pain sensations during EEG recording.

All participants underwent EEG while subjects were sitting in a dark room, fully awake but with the eyes closed. Unipolar recordings were performed from 16 electrodes (Fp1, Fp2, F3, F4, F7, F8, C3, C4, T3, T4, T5, T6, P3, P4, O1, O2) according to the International 10–20 system. Linked-ear reference was used for recording. Impedances were below 10 kΩ in all electrodes processed in the further analysis. EEG signals were registered using the “Neuro-KM21” system (analog-to-digital converter with amplifier; sampling rate, 200 Hz, 0.5–30 Hz band pass filter, notch filter at 50 Hz; Russia) and continuously viewed on a PC monitor.

Twenty minutes of EEG were recorded, including resting wakeful eyes closed state (usually 3–4 min) followed by routine activation maneuvers according to standard clinical EEG protocol. In present work, we focused our analysis on the spontaneous EEG before EEG activation, i.e., on a resting state eyes closed EEG activity since it has been shown that clinical dysfunction altered resting-state networks in MS (45). The electroencephalographer was blind to the diagnosis of the subject at the time of recording, processing, and interpretation of electrophysiological data.

### EEG Processing

EEGs were reviewed and analyzed offline with the commercial software “BRAINSYS” (Version 2.0 for Windows, Russia) (46). EEG was visually inspected, and segments contaminated with eye movements, electromyography, or other artifacts of technical origin were manually removed. Preprocessed EEG data were then processed using the BRAINSYS algorithm for advanced artifact search when the EEG amplitude is considered as a random variable, and its deviation from the zero lines by 4–5 SD serves as criteria for identifying an artifact. These marked sites were additionally inspected.

Data were segmented into 4-s epochs (without overlapping); for each participant, 15–25 free of artifact segments were included in further analyses: (mean ± SEM; 20.2 ± 1.0 epochs of 80.8 ± 4.2 s duration on the average). Epoch's number did not significantly differ between groups: 23.3 ± 2.0, 20.3 ± 1.8, and 17.4 ± 1.6 in MS group with CNP, MS group without CNP, and HC, correspondingly [*F*_(2, 33)_ = 2,740; *p* = 0.079].

Analysis of the power spectral density of electrical activity (EA) of the brain was carried out according to fast Fourier transform algorithm, in the frequencies of five physiological ranges: δ (delta, 0.5–4.0 Hz), θ (theta, 4.0–8.0 Hz), α (alpha, 8.0–13.0 Hz), β1 (beta1, 13.0–20.0 Hz), and β2 (beta2, 20.0–30.0 Hz).

In every frequency band, we identified the following characteristics: absolute power spectral density (aPSD, μV^2^), relative power spectral density (rPSD, %) defined by the ratio of the absolute power spectral density in the actual frequency band to the overall absolute spectral power computed as the sum of power spectral density' values within all empirically defined bands of interest (in percent), and peak frequency—the frequency of the dominant spectral power in the frequency band (Hz—*per se* the frequency of the amplitude maximum of spectral power in the band). Simultaneous evaluation of aPSD and rPSD increases the information content of the analysis since rPSD allows evaluating the contribution of each rhythm component to the total EEG power in the analyzed segment.

### Data Analysis and Statistics

All statistics were calculated with statistical package “STATISTICA 7.0” for Windows and “BRAINSYS” software. We tested all variables comparing between patients and controls for normal distribution by the Kolmogorov–Smirnov test. If they proved to be normally distributed, we applied the parametric tests for independent samples; otherwise, we used the non-parametric tests.

Logarithmic transformation was used for any power spectral density value to achieve a valid normal distribution of these data and allow an ANOVA analysis (47); for absolute spectral power, we calculated Ln(aPSD); for relative spectral power:

(1) $$\begin{array}{l} \text{Ln }\left(\text{rPSD}\right)=\text{Ln}\left[\left(\text{power }\left(\%\right)\right)/\left(100-\text{power }\left(\%\right)\right)\right] \end{array}$$

where Ln is the natural logarithm and power (%) is the relative spectral power (%).

Ln(absolute spectral power) and Ln[(relative spectral power)/(100 – relative spectral)] for absolute and relative spectral power, respectively. The homogeneity of variances in compared samples was verified with Bartlett's test. Differences in spectral power between groups were assessed using analysis of variance (ANOVA) followed by pairwise *post-hoc* comparison using Duncan test to account for multiple comparisons. Regional normalized power spectral density values were used as dependent variable of the ANOVAs. The three-way ANOVA factors were the following: group [levels (3): MS with CNP, MS without CNP, healthy control; independent variable], region of interest (ROI) [levels (8): prefrontal (Fp1, Fp2), frontal (F3, F4), central (C3, C4), parietal (P3, P4), occipital (O1, O2), anterior temporal (F7, F8), mid-temporal (T3, T4), and posterior temporal (T5, T6); independent variable], and hemisphere [levels (2): left and right; independent variable]. For the test statistics of ANOVA, we calculated eta-squared (η^2^) as effect size (ES) measurement using the standard formula (48, 49):

(2) $$\begin{array}{l} \eta^{2}=\text{S}{\text{S}}_{\text{Effect}}/\text{S}{\text{S}}_{\text{Total}} \end{array}$$

where SS_Effect_ is the sum of squares of effect and SS_Total_ is the total sum of squares.

ES interpretation was the following: ES ≤ 0.02 indicates a weak effect, 0.02 < ES ≤ 0.13 indicates a moderate effect, and ES > 0.13 indicates a strong effect (50). When ANOVA showed 0.050 < *p* ≤ 0.085 and ES was moderate, we considered a trend.

Differences in peak frequency between groups were estimated using Kruskal–Wallis ANOVA followed by multiple comparisons of mean ranks (two-tailed). For the test statistics of Kruskal–Wallis *H*, we calculated eta-squared (η^2^) and Cohen's *d* (*d*_Cohen_) as effect size (ES) measurement using an ES calculator (51). Cohen's *d* of 0.20–0.40 indicates a small effect, 0.50–0.70 indicates a medium-sized effect, and 0.80–1.0 and more indicates a large effect; eta-squared of 0.010–0.039 indicates a small effect, 0.060–0.110 indicates a medium-sized effect, and 0.140–0.200 indicates a large effect (51, 52). When ANOVA showed 0.050 < *p* ≤ 0.085 and Cohen's *d* was more than 0.8 or η^2^ was more than 0.140, we considered a trend.

Differences between groups (EDSS, the clinical score of neurological signs) were evaluated using Wilcoxon–Mann–Whitney rank sum test (two groups). Pearson chi-square (χ^2^ criterion) was applied to test the difference between the two groups in terms of percentages of female/male (gender index). The non-parametric Spearman's rank correlation coefficient rho (ρ_S_) with false discovery rate control (53) to correct for multiple comparisons was used for correlation analyzes. All the tests were two-tailed. The α level was set at 0.05.

## Results

### Sample Characteristics

In all the groups, neither the mean age [*F*_(2, 33)_ = 0.899; *p* = 0.416, one-way ANOVA] nor the gender index (*p* > 0.05, χ^2^ criterion) significantly differed (see Table 1). Thus, the controls were age- and gender-matched clinically normal subjects. MS groups with and without CNP were fully comparable in the frequency of the occurrence of the disease course and comorbid disorders. The mean number of years since MS diagnosis in groups with and without CNP also did not significantly differ [*F*_(1, 22)_ = 0,001; *p* = 0.982, one-way ANOVA] same as the disease severity as judged by EDSS scores (*p* = 0.843, Wilcoxon–Mann–Whitney test).

### Analysis of EEG Power Spectra

#### Analysis of Absolute Power Spectral Density

**Figure 1:**
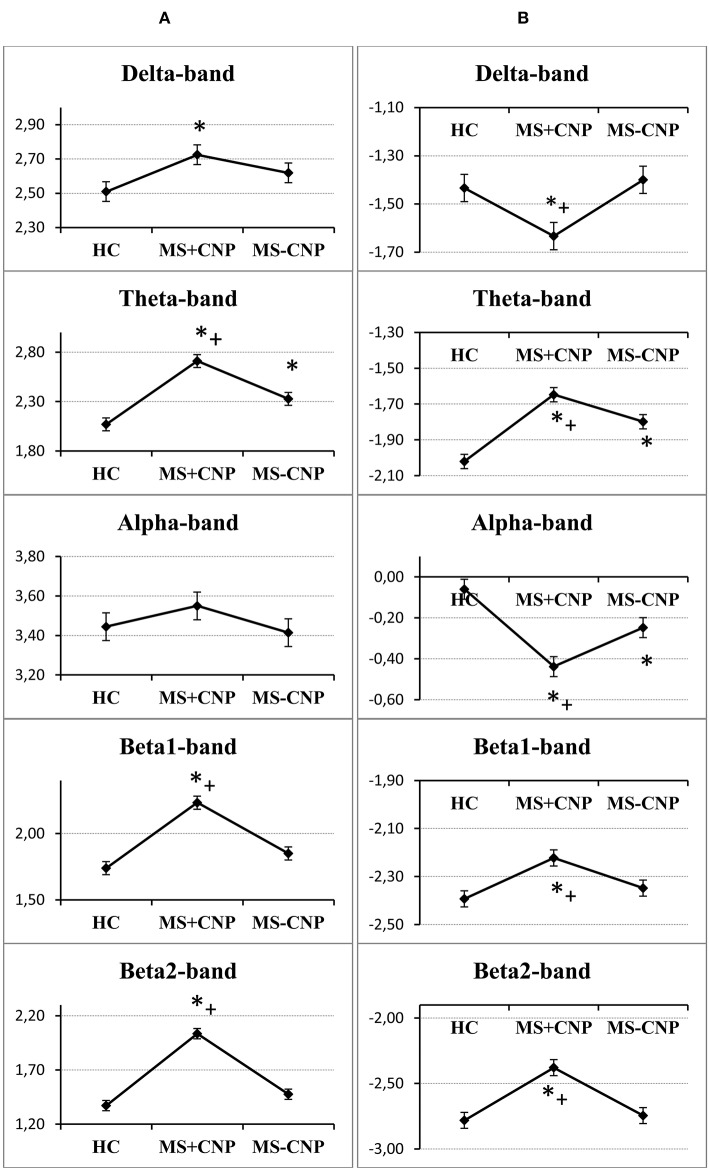
Group factor effects on the absolute and relative power spectral density in the five frequencies ranges. **(A)** A vertical row on the left side, the vertical axis on the graphics indicate normalized absolute power spectral density; **(B)** a vertical row on the right side, the vertical axis on the graphics indicate normalized relative power spectral density. HC, healthy control; MS + CNP, multiple sclerosis patients with CNP; MS-CNP, multiple sclerosis patients without CNP. *p < 0.05 as compared with the HC group; ^+^*p* < 0.05 as compared with MS patients without CNP (three-way ANOVA followed by *post-hoc* Duncan test).

**Figure 2:**
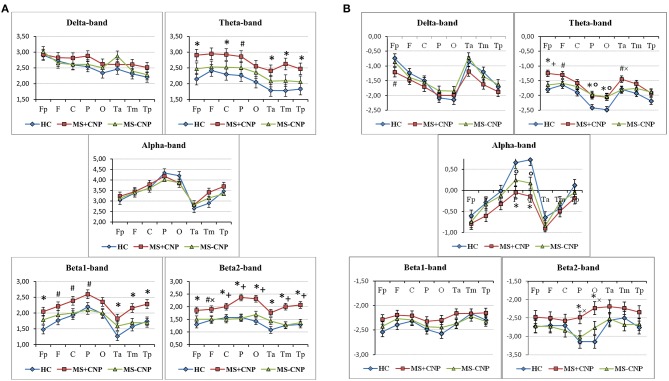
Topographical differences between the groups in the absolute **(A)** and relative **(B)** power spectral density in the five frequencies ranges. The graphics show the normalized PSD values in the following ROI: prefrontal (Fp), frontal (F), central (C), parietal (P), occipital (O), anterior temporal (Ta), mid-temporal (Tm), and posterior temporal (Tp). HC, healthy control; MS + CNP, multiple sclerosis patients with CNP; MS-CNP, multiple sclerosis patients without CNP. *p < 0.05, ^#^*p* < 0.08—comparing the MS + CNP group vs. the HC group; ^+^*p* < 0.05, ^×^p<0.08—comparing the MS + CNP group vs. MS-CNP group; *p* < 0.05—comparing the MS-CNP group vs. the HC group (three-way ANOVA followed by *post-hoc* Duncan test).

In the δ band, the three-way ANOVA showed a group effect on aPSD of the scalp EEG [*F*_(2, 528)_ = 3.501, *p* = 0.031; η^2^ = 0.012]. Only in MS with CNP group, absolute spectral powers significantly increased as compared with HC: according to the *post-hoc* Duncan test, *p* = 0.011 (Figure 1A). The main effect of the ROI factor was significant [*F*_(7, 528)_ = 4.177, *p* < 0.001; η^2^ = 0.051] and revealed higher aPSD in the prefrontal region as compared with occipital, posterior, and mid-temporal regions (in all cases, *p* < 0.05) with the least power in the posterior temporal region. However, we did not observe significant differences between aPSD in any of the ROIs (Figure 2A) and derivations in MS groups and HC. The hemisphere factor did not affect aPSD either in the δ band or in the other frequency ranges studied.

**Figure 3:**
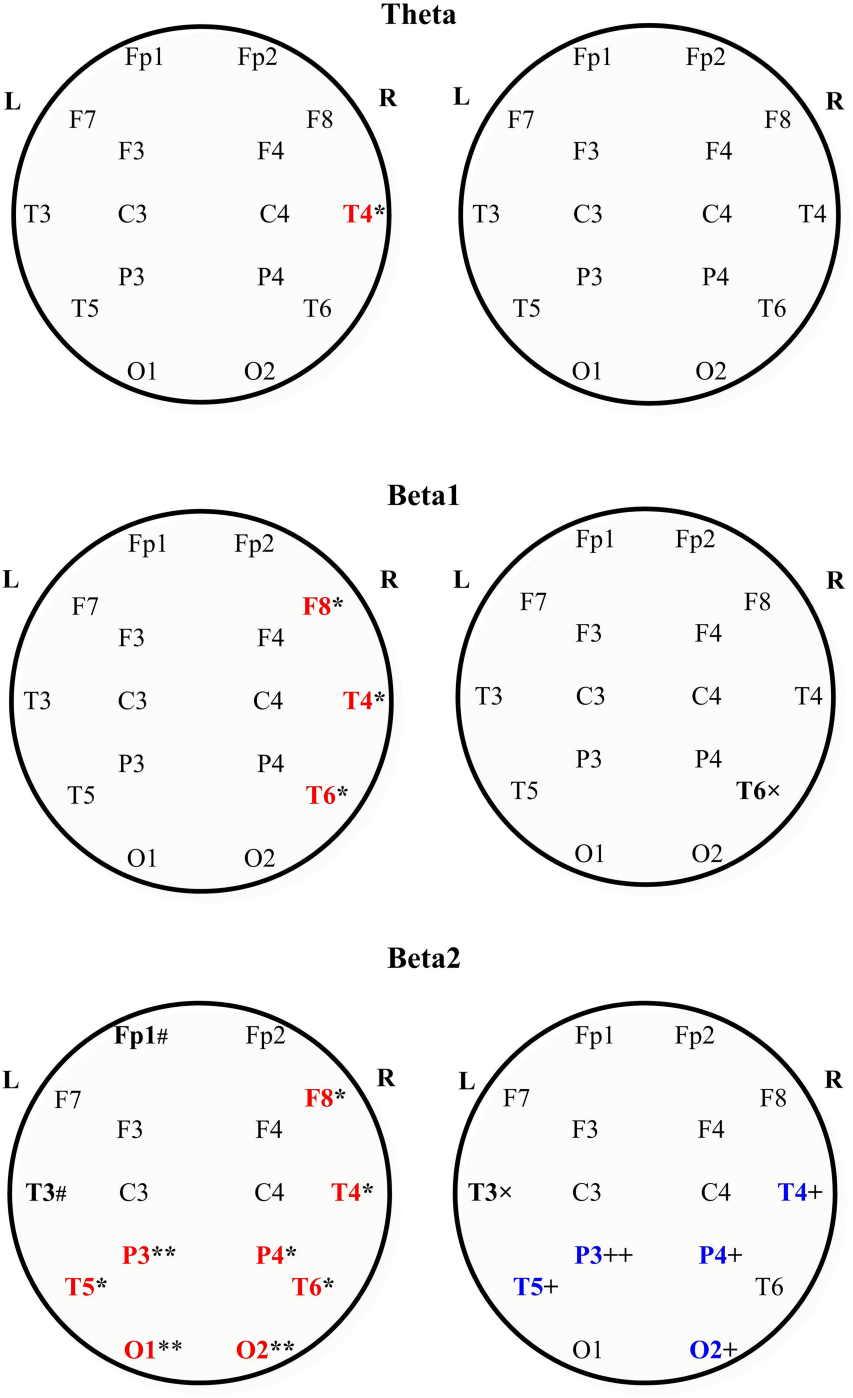
Absolute power spectral density of the scalp EEG in the theta (the upper row), beta1 the middle row), and beta2 bands (the bottom row) in MS patients with and without central neuropathic pain. Topographic view of each scalp electrode positions used in this study. Heads are shown in top view. L, left hemisphere; R, right hemisphere. In each row: MS with CNP—to the left; MS without CNP—to the right. The large bold letters represent the following: red—the channels where the absolute spectral power increased as compared with control; blue—the channels where the absolute spectral power decreased as compared with MS patients with CNP; black—the channels where a trend toward difference with one of the groups was revealed. Boundaries of frequency ranges, Hz; see Materials and Methods; *n* = 12 for each group. *post-hoc* results for spectral analysis of the frequency bands (Duncan test) are shown. Statistically significant differences: *p < 0.05; **p < 0.01 as compared with control; ^+^*p* < 0.05; ^++^*p* < 0.01 as compared with MS patients with CNP. # Indicates a trend (0.05 < *p* < 0.06) to increase as compared with control; × indicates a trend (0.05 < *p* < 0.06) to decrease as compared with MS with CNP. See the Supplementary Table for more information.

In the θ band, aPSD in both MS groups increased as compared with HC [group factor: *F*_(2, 528)_ = 24.372, *p* < 0.001, three-way ANOVA; η^2^ = 0.080; in both cases, *p* < 0.01] (Figure 1A). Besides, power density in MS with CNP group exceeded that in MS without CNP group (*p* < 0.001, Duncan test). ROI factor [*F*_(7, 528)_ = 4.349, *p* < 0.001; η^2^ = 0.050] showed the decreased power density in the temporal regions as compared with the other areas except for the occipital cortex (in all cases, *p* < 0.05, Duncan test). MS patients with CNP demonstrated increased absolute power density for the prefrontal, central, parietal (a trend), and all temporal regions as compared with the HC group (Figure 2A). Besides, we revealed higher aPSD for the mid-temporal derivation of the right hemisphere (T4) as compared with the control (results of *post-hoc* analysis are presented in Figure 3). We saw no difference between MS patients with and without CNP or MS patients without CNP and HC in any ROI and derivation.

In the α band, ANOVA showed no group effect on EEG aPSD [*F*_(2, 528)_ = 1.026, NS; η^2^ = 0.003] (Figure 1A). ROI effect was significant [*F*_(7, 528)_ = 16.490, *p* < 0.001; η^2^ = 0.175] that expectedly manifested as a higher EEG power in the occipital and parietal regions compared with other brain regions. Between-group differences were not revealed (Figure 2A).

In the β1 band, only MS patients in group with CNP showed increased EEG aPSD compared with HC and MS patients without CNP as accounted by the ANOVA main effect group factor [*F*_(2, 528)_ = 27.635, *p* < 0.001; η^2^ = 0.084; in both cases; Duncan test, *p* < 0.001] (Figure 1A). ANOVA main effect for ROI factor was significant [*F*_(7, 528)_ = 8.332, *p* < 0.001; η^2^ = 0.089]: in anterior temporal region, aPSD was lower than in frontal, central, parietal, and occipital regions; also, in prefrontal and mid-temporal regions, it was lower than in central, parietal, and occipital regions (in most cases, *p* < 0.001). *Post-hoc* Duncan test revealed differences in power spectral density between the groups. MS patients with CNP showed a statistically significant increase in aPSD in prefrontal and all temporal regions and a trend to increase aPSD in frontal, parietal, and occipital regions as compared with the HC group (Figure 2A). In addition, in the posterior (T6), mid- (T4), and anterior temporal derivations (F8) of the right hemisphere, the spectral power in MS with CNP group was higher than in HC (see Figure 3). In the posterior temporal derivation (T6), absolute power in MS patients without CNP tended (*p* = 0.059) to be lower than in MS patients with CNP. We did not reveal any statistically significant differences for this band between MS without CNP and HC groups.

In the β2 band, similar to the β1 band, EEG aPSD increased in MS with CNP group compared with MS without CNP group and HC [ANOVA main effect for group factor: *F*_(2, 528)_ = 56.856, *p* < 0.001; η^2^ = 0.167; in both cases, Duncan test: *p* < 0.001] (Figure 1A). ANOVA showed a significant ROI effect [*F*_(7, 528)_ = 3.374, *p* = 0.002; η^2^ = 0.035]: in anterior temporal region, power density was lower than in the central, parietal, and occipital regions (in most cases, *p* < 0.05, Duncan test). In the most ROIs, aPSD increased as compared with HC (Figure 2A); also, in central, parietal, occipital, mid-, and posterior temporal regions, absolute power density was higher than in the MS without CNP group. In MS with CNP group, we noted an upward trend for aPSD in the frontal region compared with the HC and MS without CNP groups. In the cortical derivations (excluding central, frontal, and prefrontal in both hemispheres and anterior temporal derivation in the left hemisphere), spectral power exceeded the corresponding values in the HC group (see Figure 3). In the mid- and posterior temporal, parietal, and occipital derivations, EEG aPSD in MS without CNP group was lower than in MS with CNP group (except for the occipital derivation of the left hemisphere and the posterior temporal derivation of the right hemisphere). Similar to the other frequency bands, aPSD in MS patients without CNP did not significantly differ from the HC group in any region and derivation.

ANOVA showed no statistically significant interaction effect on EEG aPSD for group × ROI, group × hemisphere, ROI × hemisphere, and group × ROI × hemisphere in any frequency band.

#### Analysis of Relative Power Spectral Density

In the δ band, EEG rPSD decreased in MS with CNP group compared with HC and MS without CNP groups as accounted by the ANOVA main effect group [*F*_(2, 528)_ = 4.979; *p* = 0.007; η^2^ = 0.014; according to *post-hoc* Duncan test, *p* = 0.013 and *p* = 0.005, respectively] (Figure 1B). Relative power density increased from the posterior areas of the cortex to the anterior regions as accounted by the ANOVA main effect ROI [*F*_(7, 528)_ = 20.333; *p* < 0.001; η^2^ = 0.206]. We did not observe significant differences between rPSD in any of the ROIs (Figure 2B) and derivations in MS groups and HC. The δ-band rPSD showed no significant main effect for hemisphere [*F*_(1, 528)_ = 0.210; NS].

**Figure 4:**
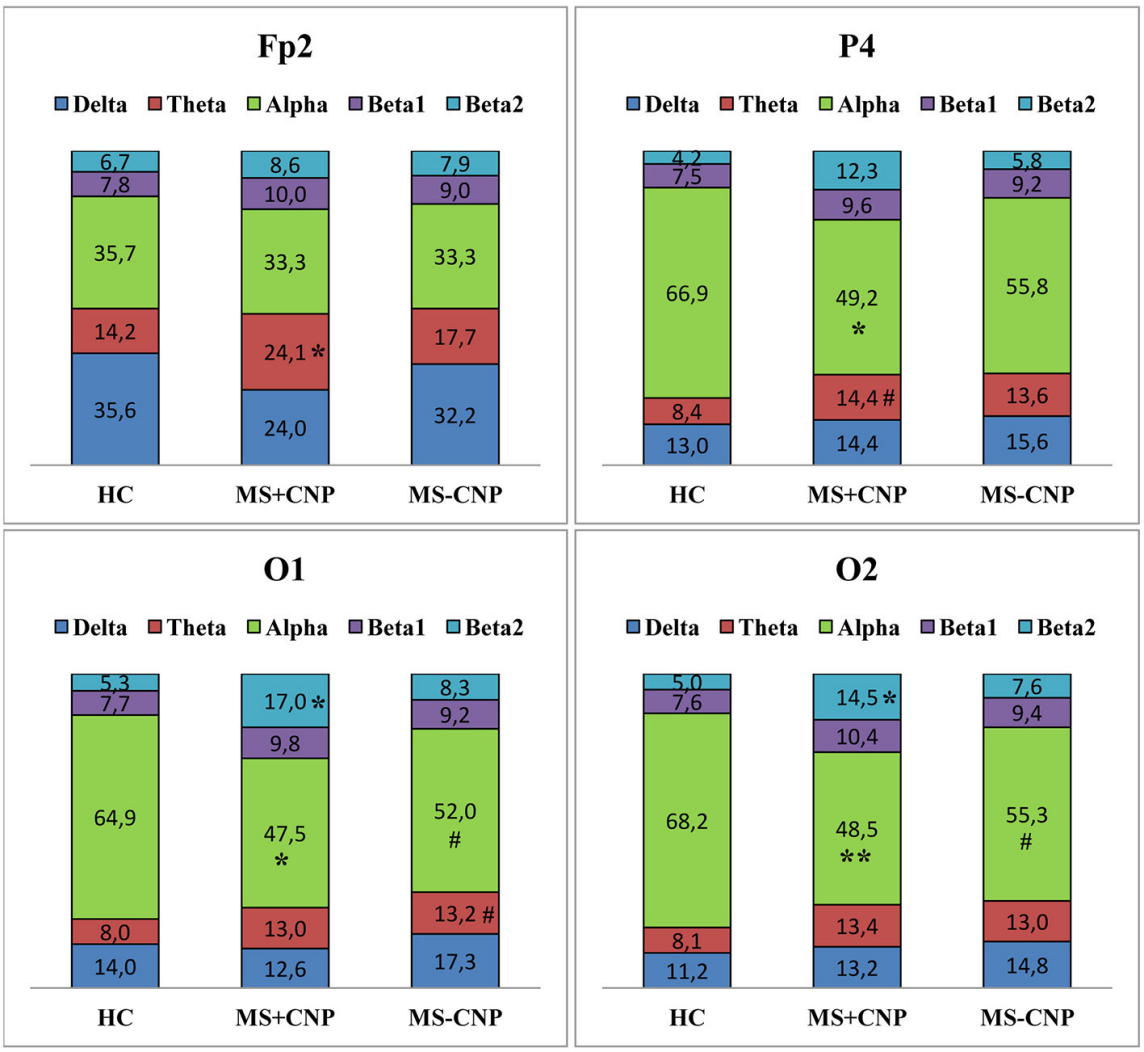
Relative power spectral density (%) of the scalp EEG for Fp2, P4, O1, and O2 electrodes positions in MS patients with and without central neuropathic pain. HC, healthy control; MS + CNP, multiple sclerosis patients with CNP; MS-CNP, multiple sclerosis patients without CNP. *Post-hoc* results for spectral analysis of the frequency bands (Duncan test) are shown. Statistically significant differences: *p < 0.05; **p < 0.01 as compared with control; # indicates a trend (0.05 < *p* ≤ 0.084) as compared with control. See the Supplementary Table for more information.

In the θ band, rPSD, similar to aPSD, increased in both MS groups as compared with HC [ANOVA main effect group factor: *F*_(2, 528)_ = 22.146, *p* < 0.001; η^2^ = 0.064; in both cases, *p* < 0.001] (Figure 1B). Power density in MS with CNP group also exceeded that in MS without CNP group (*p* = 0.007). Three-way ANOVA main effect for ROI factor [*F*_(7, 528)_ = 15.668, *p* < 0.001; η^2^ = 0.158] showed decreased rPSD in posterior areas compared with central, prefrontal, and frontal regions; in posterior temporal region, rPSD were lower than in anterior and mid-temporal regions (in most comparisons, *p* < 0.01, Duncan test). In the prefrontal region, rPSD in MS with CNP patients was higher than in the HC group and stronger than in MS without CNP patients (Figure 2B). In the anterior temporal region, a trend toward an increase in rPSD was observed in the MS with CNP group compared with the other groups. In parietal and occipital regions, spectral power exceeded control values in both MS groups. Between groups, a statistically significant difference was only found for the anterior frontal derivation in the right hemisphere (Fp2): the relative power density in MS patients with CNP was higher than that in the HC group (Figure 4). In MS without CNP group, *post-hoc* Duncan test showed a trend to increase rPSD as compared with HC in the occipital derivations of the left hemisphere (*p* = 0.082). There were no significant differences in rPSD in any of the derivations between MS groups with and without CNP. The θ-band rPSD showed no significant main effect for hemisphere [*F*_(1, 528)_ = 0.090; NS].

In the α band, in contrast, a decrease in relative spectral power in MS groups with and without CNP as compared with HC was found as accounted by the ANOVA main effect group [*F*_(2, 528)_ = 15.033; *p* < 0.001; η^2^ = 0.040; according to Duncan test, *p* < 0.001 and *p* = 0.007, respectively] (Figure 1B). In MS patients with CNP, relative PSD also was lower than in MS patients without CNP (*p* = 0.006, Duncan test). ROI effect was significant and strong [*F*_(7, 528)_ = 25.973; *p* < 0.001; η^2^ = 0.239]: power density, as expected, was decreasing toward the frontal areas. In both MS groups, rPSD in parietal and occipital regions decreased as compared with the HC group (Figure 2B). However, the detailed analysis showed that only in MS patients with CNP, rPSD was significantly less than in HC in the occipital derivations of both hemispheres and parietal lobe of the right hemisphere (see Figure 4), whereas in MS patients without CNP, rPSD in occipital derivations only showed a trend to decrease as compared with the control (for O1, *p* = 0.082; for O2, *p* = 0.084, Duncan test). MS groups with and without CNP did not statistically differ from each other in any ROIs and derivations. The α-band rPSD showed no significant main effect for hemisphere [*F*_(1, 528)_ = 1.550; NS].

In the β1 band, the main effect of the group factor was found [*F*_(2, 528)_ = 6.937; *p* = 0.001; η^2^ = 0.024]. According to Duncan test, relative PSD in MS with CNP group exceeded power density in HC and MS without CNP group: *p* < 0.001 and *p* = 0.008, respectively (Figure 1B). ANOVA main effect for ROI factor [*F*_(7, 528)_ = 2.761, *p* = 0.008; η^2^ = 0.034] showed the strongest power in the mid-temporal region as compared with prefrontal and posterior areas (in most comparisons, *p* < 0.01, Duncan test). MS groups did not differ between themselves in any ROIs (Figure 2B) and derivations. The β1-band rPSD showed no significant main effect for hemisphere [*F*_(1, 528)_ = 0.012; NS].

In the β2 band, similar to the effect in β1 band, rPSD in MS patients with CNP increased as compared with HC (*p* < 0.001) and MS patients without CNP (*p* < 0.001) as accounted by the ANOVA main effect group [*F*_(2, 527)_ = 13.293; *p* < 0.001; η^2^ = 0.045] (Figure 2B). In addition, the main effect for ROI factor was statistically significant [*F*_(7, 527)_ = 2.116; *p* = 0.040; η^2^ = 0.026] with the least power in parietal region. Relative PSD in the parietal and occipital regions was stronger in MS with CNP patients as compared with the control and tended to exceed rPSD in MS patients without CNP (Figure 2B). Moreover, in MS with CNP group, relative PSD in both occipital derivations (O1 and O2) exceeded control values (see Figure 4). The β2-band rPSD showed a significant although weak ANOVA main effect for hemisphere [*F*_(1, 527)_ = 2.116; *p* = 0.040; η^2^ = 0.026] with the stronger power in the left hemisphere (*p* = 0.039, Duncan test). In MS with CNP group, rPSD in both hemispheres was stronger than in the other groups: in all cases (*p* < 0.05, Duncan test).

ANOVA did not show any statistically significant interaction effect on EEG rPSD for group × ROI, group × hemisphere, ROI × hemisphere, and group × ROI × Hemisphere in any frequency band.

### Analysis of Peak Frequency

Table 2 shows the changes in peak frequency. In the δ band, in the MS with CNP group, peak frequencies in the left frontal area (F3) were higher than in the control group. In the MS without CNP group, in contrast, peak frequency in a posterior temporal derivation of the left hemisphere (T5) was lower than in the control group. Peak frequency in MS patients without CNP was lower than in MS patients with CNP in a variety of derivations—the right posterior temporal (T6) and both mid-temporal (T3, T4), the right frontal (F4) and occipital (O2), and both central (C3, C4) derivations.

**Table 2:** Peak frequency (Hz) in the δ and β2-bands in patients with multiple sclerosis.

**Electrodes**	**Results of Kruskal-Wallis ANOVA** **H (2**, ***N*** **=** **36);** ***p***		**Effect size** **η**^**2**^**; d**_**Cohen**_		**Healthy control**		**MS with CNP**		**MS without CNP**	
	**δ**	**β_2_**	**δ**	**β_2_**	**δ**	**β_2_**	**δ**	**β_2_**	**δ**	**β_2_**
O1	5.72; 0.06	7.72; 0.02	0.113; 0.713	0.173; 0.916	1.5 (0.8)	21.4 (2.0)	2.2 (0.8)	24.6 (2.5)^*^	1.8 (0.9)	22.6 (2.5)
O2	7.81; 0.02	5.61; 0.06	0.176; 0.924	0.109; 0.701	1.7 (0.8)	21.8 (2.3)	2.0 (0.7)	24.2 (2.5)	**1.4 (1.0)**^**+**^	22.4 (2.8)
P3	5.20; 0.07	9.92; 0.01	0.097; 0.655	0.240; 1.124	2.0 (1.1)	21.5 (2.3)	2.3 (0.8)	25.2(1.8)^**^	1.5 (0.8)	22.6 (2.5)
P4	1.63; 0.44	0.02; 0.99	0.011; 0.214	0.060; 0.505	1.9 (0.9)	22.0 (2.5)	2.1 (0.8)	22.7 (2.8)	1.8(0.9)	22.0 (2.2)
C3	7.35; 0.03	0.16; 0.92	0.162; 0.880	0.056; 0.486	1.8 (0.8)	22.2 (2.5)	2.3 (0.7)	22.9 (2.7)	**1.6 (0.7)**^**+**^	22.6 (2.5)
C4	10.78; 0.00	0.35; 0.84	0.266; 1.204	0.050; 0.460	1.8 (0.8)	22.1 (2.6)	2.4 (0.7)	22.5 (2.7)	**1.3 (0.6)**^**++**^	21.2 (1.6)
F3	6.89; 0.03	4.16; 0.12	0.148; 0.834	0.066; 0.529	1.6 (0.8)	22.2 (2.4)	2.3 (0.7)^*^	24.3 (2.4)	1.7 (1.0)	22.1 (2.3)
F4	6.83; 0.03	0.35; 0.84	0.146; 0.828	0.050; 0.459	1.6(0.9)	21.9 (2.3)	2.3 (0.7)	22.9 (2.8)	**1.5 (1.1)**^**+**^	22.0 (2.0)
Fp1	5.21; 0.07	4.00; 0.14	0.097; 0.656	0.061; 0.508	1.2 (0.6)	22.2 (2.6)	1.6 (0.5)	24.4 (2.4)	1.3 (0.9)	22.4 (2.6)
Fp2	3.63; 0.16	1.19; 0.55	0.049; 0.455	0.024; 0.316	1.4 (0.5)	22.7 (3.2)	1.9 (0.9)	23.4 (2.7)	1.3 (0.4)	21.6 2.0
T5	6.57; 0.04	7.06; 0.03	0.138; 0.802	0.153; 0.851	2.1 (0.9)	22.4 (2.4)	1.9 (0.7)	24.7 (2.1)^*^	1.3 (0.6)^*^	22.7 (2.4)
T6	6.10; <0.05	0.91; 0.64	0.124; 0.753	0.033; 0.370	1.5 (0.9)	22.3 (2.4)	2.1 (0.9)	23.2 (2.8)	**1.3 (0.5)**^**+**^	22.2 (2.3)
T3	6.26; 0.04	2.21; 0.33	0.129; 0.770	0.006; 0.161	1.7 (0.8)	22.4 (2.2)	1.9 (0.7)	24.0 (2.6)	**1.2 (0.6)**^**+**^	22.4 (2.6)
T4	10.95; 0.00	0.09; 0.95	0.271; 1.220	0.058; 0.495	1.6 (0.9)	23.0 (2.6)	2.2 (0.7)	23.0 (2.7)	**1.2 (0.4)**^**++**^	22.4 (2.2)
F7	4.00; 0.14	6.73; 0.03	0.061; 0.508	0.143; 0.818	1.3 (0.6)	22.6 (3.2)	1.7 (0.7)	**25.0 (2.1)**^&^	1.2 (0.4)	22.7 (2.4)
F8	0.35; 0.84	3.86; 0.15	0.050; 0.459	0.056; 0.488	1.3 (0.6)	22.5 (3.1)	1.3 (0.7)	24.1 (2.3)	1.4 (0.8)	22.6 (2.1)

*Peak frequency is shown as Mean (Standard Deviation)*.

* p < 0.05;

** p < 0.05;

& p = 0.052 as compared with ≪Control≫;

+ p < 0.05;

++ *p < 0.01 as compared with ≪MS with CNP≫ by multiple comparisons of mean ranks (2-tailed). Statistically significant differences are shown in bold*.

In the β2 band, in the MS with CNP group, peak frequencies in the occipital, parietal, and posterior temporal derivations of the left hemisphere (O1, P3, and T5) were higher than in the control group. In addition, we saw a strong tendency to increase peak frequency for the frontal derivation of the left hemisphere (F7) in comparison with the control group.

No changes of peak frequency were detected in the other frequency bands.

### Analysis of Correlations

We failed to reveal any statistically significant correlations between absolute spectral power in the five frequency bands and the intensity of pain on the VAS scale in MS patients with CNP.

We also did not find any correlation between absolute or relative spectral power for any derivations in the frequency bands and EDSS score in MS patients with and without CNP.

## Discussion

The present study used a new approach to the analysis of EEG peculiarities in patients with MS, based on whether they have CNP. Although the theta band absolute PSD showed an increase in MS patients both with and without CNP (see Figure 1A), the power in the theta as well as beta1, and beta2 ranges in MS with CNP group exceeded that in patients without pain. Only MS patients with CNP demonstrated the significantly increased absolute spectral powers for the theta, beta1, and beta2 frequency bands in most regions of interest (see Figure 2A). Moreover, in the beta2 frequency range, the power exceeded that in patients without pain in almost all the regions examined. Detailed analysis of derivations showed the peculiarities of the differences between MS patients with and without CNP: only MS patients with CNP demonstrated the increased absolute spectral powers for the theta and beta1 frequency bands in the temporal lobes of the right hemisphere, and the increased powers for the beta2 band in the occipital, parietal, and temporal lobes of both hemispheres (see Figure 3).

On the basis of EEG and functional low-resolution electromagnetic tomography, peak overactivation mainly within the theta (6–9 Hz) and low beta frequency bands (12–16 Hz) localized to multiple pain-associated areas (primarily to insular, anterior cingulate, prefrontal, and inferior posterior parietal cortices, as well as to primary, secondary, and supplementary somatosensory cortices) and slight overactivation in the high beta frequency band (16–30 Hz) localized to the occipital lobe have been reported for neurogenic pain patients (38). Only using EEG, we observed similar overactivation for the theta (4.0–8.0 Hz) and beta1 (13.0–20.0 Hz) bands in the right hemisphere and the beta2 (20.0–30.0 Hz) band in the posterior areas (including occipital lobes) in MS patients with CNP. Of interest, the main changes in theta and low beta bands frequency ranges were found in the right hemisphere, although there was an equal number of patients with pains on the left and right sides in the group. Below, we consider the possible ways and mechanisms of the revealed EEG changes.

Vazquez-Marrufo et al. (28) identified the increase in high-frequency bands [for beta (22–30 Hz) and gamma (31–45 Hz), not for low beta (13–21 Hz) or theta (5–8 Hz) bands] in both the occipital regions (O1 and O2) and the frontal right hemisphere region (F4) in the relapsing–remitting multiple sclerosis (RRMS) group of patients when the subjects were being stressed during a visuospatial task. Although the authors did not report neuropathic pain in patients, we assume CNP to be a stress factor, the effect of which reflects in the increment of high bands in the EEG.

The increment of high bands could be caused in different ways, for example, by the increase in anxiety (54) [especially in the central part of frontal cortex (55)], by administration of some psychotropic drugs (56, 57) or by physiological artifacts—due to muscle activity in temporal lobes (28). In our research, MS patients with and without CNP did not differ in anxiety (58, 59). All included patients were free of any psychotropic medication during the participation in the study at least for a month. Besides, the changes in the high-frequency band were observed not only in temporal but also in occipital, parietal, central, frontal, and prefrontal regions, which allows discarding muscle influence. Thus, the increment of the beta2 band might be an additional indicator for CNP in MS patients.

The lack of correlation between the degree of disability score and EEG values in any of the brain areas and spectral power bands in MS patients with and without CNP in the present study in principle agreed with the results reported by the other authors for MS patients, regardless CNP (26, 28). Thus, it is unlikely that the overactivation of beta and theta bands in the cortex associated with neurological impairment.

ANOVA revealed the decrease in relative power spectral density in the alpha band in MS patients in posterior areas regardless of the pain. The effect was stronger in MS with CNP group (see Figures 1B, 2B). This finding is in good agreement with the data of Babiloni et al. (60), who demonstrated abnormal cortical sources of resting state in MS patients with both RRMS and SP subtypes compared to the HC group: increase in delta (higher amplitude) and decreased in alpha (lower amplitude) rhythms as estimated by LORETA (normalized relative current density at the cortical voxels). Decrement in the relative spectral power in the alpha band in the posterior brain areas of the right hemisphere, which we identified with a detailed analysis in MS patients with CNP, may testify to a reorganization of the brain EA, which appears as the increase in spectral power in the theta and beta bands in the right hemisphere. This assumption conforms to functional MRI exploration, where patients at the earliest stage of MS showed significantly higher activation in the right frontopolar cortex, the bilateral lateral prefrontal cortices, and the right cerebellum during a cognitive task (13). Lenne et al. (61) found a significant decrease in mutual information in a network of brain areas in patients with RRMS during resting condition. Interhemispheric and right hemisphere mutual information was significantly lower in patients with MS than in control subjects that can reflect the global disconnection of cortico-cortical or cortico-subcortical areas in RRMS. However, the authors did not report whether any patients suffered from CNP.

In the present study, the clinical course of the disease was defined as RRMS in most patients, and there were no cognitively deficient patients according to Mini-Mental State Examination in both experimental groups. However, only MS patients with CNP showed the increase in EA power in the theta band and the low beta band for temporal lobes of the right hemisphere as well as a significant decrease in relative power spectral density in the alpha band in the occipital region.

According to the concept of thalamocortical dysrhythmia, the chronic neurogenic pain mechanism may be triggered at thalamic levels when a decreased excitatory input into the thalamus results in a shifted mode of thalamocortical processing, consisting of a functionally disconnected rhythmic activity at both slow (theta, 4–9 Hz) and fast (beta, 12–30 Hz/gamma, 30–80 Hz) rhythms (33, 36–38). In experiments, Hughes and Crunelli (62) demonstrated that the thalamus could act as an independent pacemaker of α and θ rhythms. Thus, the shifts in the EEG revealed in our study, namely, a decrease in PSD in the alpha band and, on the contrary, an increase in PSD in the theta band, could associate with thalamic deactivation (dysfunction). We said above that all the examined patients were right-handed subjects. Recent MR studies have revealed neuroanatomical features and specifics of the cerebral asymmetry in right-handers. Barrick et al. (63) showed gray matter rightward asymmetry of the thalamus and inferior parietal lobe. MRI diffusion tensor imaging tractography revealed a lateralized right-sided upper brainstem–thalamic function as part of the dominant right-sided cortical/subcortical vestibular system in healthy right-handed subjects (64). Right-lateralized white matter connectivity between the temporoparietal junction and insula was found in the right-handers (65). The authors suggested that disruption of the temporoparietal junction–insula pathway in the right hemisphere affects the salience system in persons with chronic pain. We suggest that the dysfunction in pain connectome-including areas (e.g., thalamus, insula, inferior parietal lobe) in the right-handed MS patients with CNP may manifest by the EEG changes in the right hemisphere.

As for the QEEG analysis in the alpha band, MS patients with and without CNP had no statistically significant difference for the absolute power spectral density and peak frequency compared to each other and the HC group in any regions of the scalp. The findings are contrary to the data of Kim et al. (66) who showed the alpha peak in the EEG to be reduced or not present in MS patients with neuropathic pain: alpha peak frequencies were lower than in the age-matched healthy control within the thalamus and the posterior insula and in the posterior cingulate cortex of the default mode network (the pain connectome-including areas). In previous studies of the other authors, the “slowing down” phenomenon manifesting as a shift toward a lower dominant frequency in patients with neuropathic pain was also noted (36, 67). What could the reason be for the lack of a difference in the alpha band in our study? One of the explanations comes from the early research that has demonstrated significant improvement in the clinical state of the patients with MS and a marked increase in the mean alpha frequency in the parietooccipital region after short intensive immunosuppressive therapy (27). In our study, all the patients, including those with CNP, had previously received corticosteroid therapy that resulted in neurological improvement and could consequently normalize alpha activity. In addition, we cannot exclude that RRMS subtype affects EEG changes that primarily relate to CNP.

Of note, with increasing relative PSD in the β2 band in the left hemisphere, β2-peak frequencies increased in the left hemisphere only in MS patients with pain. To interpret the facts mentioned above, we can use the findings of the study, which shows that the left hemisphere closely relates to desynchronizing mesencephalic structures, whereas the right hemisphere, on the contrary, refers to synchronizing diencephalic brain structures (68). Authors assumed that, in a case of CNS disorder, functional state of diencephalon and brainstem structures would determine the role of each hemisphere in compensatory processes. In our studies, the changes in peak frequency in the high beta band in the left hemisphere may provide an additional indicator of disorders of the cortico-subcortical integration in MS patients with CNP and testify for the mid-stem dysfunction. In these patients, the modification of EA in the right hemisphere could be a compensatory response. In general, changes in a resting-state EEG in MS with CNP could reflect the disturbances in cortical communication. This suggestion partly conforms to the data on the decrease in mutual information in brain EA in patients with RRMS (61). Authors suppose that averaged interhemispheric mutual information obtained in a resting state is a marker for the neurological damage induced by RRMS. Meanwhile, in our study, we saw the signs of putative alterations in cortical communication only in patients with CNP.

Remarkably, spectral power in MS patients with CNP differed from the control and rarely from MS patients without CNP. Spectral power of EA in MS patients without CNP did not significantly differ from the control except for the group effect on absolute and relative PSD in the theta band and group and ROI effects on relative PSD in the alpha band. The fact is that EEG in MS patients without central neuropathic pain tended to change similarly to EEG in MS patients with CNP in the theta and alpha ranges (see Figures 2, 4) but hardly ever reached the significant difference from the other groups. Thus, spectral power in MS patients without CNP occupy an intermediate position between spectral power in MS patients with CNP and the control. We should keep in mind that all of the patients completed the course of treatment before testing and showed significant improvement in suppressing both MS symptoms and neuropathic pain. Several studies reported more severe MS, as assessed by EDSS in patients with neuropathic pain (8, 69). In the present study, in MS patients with and without CNP, EDSS score did not differ, which confirms clinical improvement. Significant EA changes in MS patients with CNP assume to consider central neuropathic pain as a stressful factor that enhances spectral power (not peak frequency) alterations typical for MS.

We have previously observed the increase in absolute spectral power in the high-frequency bands in other types of pathology, associated with a malfunction in the nervous regulation, for example, in patients with brain–gut dysregulation burdened by CNP (70, 71). We cannot exclude that the beta bands increment in the EEG might be a marker of CNS pathology that appears as neuropathic and psychosomatic disorders. The difference between spectral EEG patterns and peak frequencies in MS patients with and without CNP could represent CNS alterations related to central neuropathic pain in MS patients.

However, a small number of patients in the groups with the well-known high variability of MS courses are a potential limitation of the study. Another limitation lies within EEG recording after the course of corticosteroids in MS patients, although the period after corticosteroid withdrawal was at least a month. We could not find direct experimental evidence that corticosteroid therapy can affect the main EEG frequencies. However, polysomnographic recordings showed that during prolonged treatment with corticosteroids (for 10 days), early RRMS patients demonstrated several sleep–EEG alterations, in particular, changes in REM sleep, slow-wave sleep, and some others (72). Among the limitations of this study, we also consider a small number of EEG electrodes and a low number of the epochs analyzed. A sound conclusion requires further research.

## Data Availability Statement

All datasets generated for this study are included in the article/Supplementary Material.

## Ethics Statement

This study was carried out in accordance with the recommendations of the Ethical Committee of the Institute of General Pathology and Pathophysiology (the project approval protocol Number 5 of November 25, 2016) and was approved by the Ethical Committee (final approval protocol Number 1a of April 03, 2018). All participants signed informed consent after a complete explanation of the study in accordance with the Helsinki Declaration of 1964 with all subsequent amendments.

## Author Contributions

NK: substantial contributions to acquisition of data, to analysis and interpretation of data. MC: substantial contributions to acquisition of data, to analysis, and drafting the article. MK: substantial contributions to conception and design, revising the article critically for important intellectual content. NY: substantial contributions to conception and design, final approval of the version to be published.

### Conflict of Interest

The authors declare that the research was conducted in the absence of any commercial or financial relationships that could be construed as a potential conflict of interest.
