# Supplementary material for: Central Neuropathic Pain and Profiles of Quantitative Electroencephalography in Multiple Sclerosis Patients
Source: Front Neurol. 2020 Jan 21;10:1380. doi: 10.3389/fneur.2019.01380 (PMC6990108; doi:10.3389/fneur.2019.01380)
Supplement: Supplementary file 1 [file Table_1.DOCX]

**Supplementary Table** (the results of *post hoc* analysis are shown in the **Fig. 3** and **Fig. 4**). Absolute and relative power spectral density of electrical activity of the brain (M ± S.E.M.) in multiple sclerosis (MS) patients with and without the central neuropathic pain (CNP), and healthy control in the δ (delta: 0.5-4.0 Hz), θ (theta: 4.0-8.0 Hz), a (alpha: 8.0-13.0 Hz), β1 (beta1: 13.0-20.0 Hz), β2 (beta2: 20.0-30.0 Hz) physiological ranges. Region of Interest: Fp – pre-frontal (Fp1, Fp2), F – frontal (F3, F4), C – (C3, C4), P – parietal (P3, P4), O – occipital (O1, O2), Ta – anterior temporal (F7, F8), Tm – mid-temporal (T3, T4), and Tp – posterior temporal (T5, T6).

| Group | Hemisphere | Region  of Interest | Absolute power spectral density, µV^2^ | Relative  power spectral density, % | N |
| --- | --- | --- | --- | --- | --- |
| Delta-band | | | | | |
| Healthy Control | L | Fp | 25,434±5,220 | 33,784±4,156 | 12 |
| Healthy Control | L | F | 22,879±5,079 | 25,331±3,524 | 12 |
| Healthy Control | L | C | 20,227±4,247 | 21,755±3,080 | 12 |
| Healthy Control | L | P | 18,868±4,762 | 13,985±2,129 | 12 |
| Healthy Control | L | O | 15,558±3,336 | 13,972±2,408 | 12 |
| Healthy Control | L | Ta | 19,476±6,101 | 32,682±4,733 | 12 |
| Healthy Control | L | Tm | 14,706±4,073 | 25,756±4,182 | 12 |
| Healthy Control | L | Tp | 13,841±3,935 | 20,499±3,911 | 12 |
| Healthy Control | R | Fp | 29,595±6,394 | 35,591±4,568 | 12 |
| Healthy Control | R | F | 22,264±5,539 | 24,479±3,207 | 12 |
| Healthy Control | R | C | 19,641±4,047 | 20,282±2,789 | 12 |
| Healthy Control | R | P | 21,101±6,265 | 13,034±2,517 | 12 |
| Healthy Control | R | O | 15,294±3,499 | 11,174±1,535 | 12 |
| Healthy Control | R | Ta | 19,317±5,557 | 32,012±4,408 | 12 |
| Healthy Control | R | Tm | 21,069±6,886 | 28,106±4,998 | 12 |
| Healthy Control | R | Tp | 18,932±7,239 | 17,762±3,254 | 12 |
| MS with CNP | L | Fp | 20,858±3,285 | 24,787±3,601 | 12 |
| MS with CNP | L | F | 17,285±2,125 | 19,725±2,641 | 12 |
| MS with CNP | L | C | 17,764±2,234 | 17,634±2,711 | 12 |
| MS with CNP | L | P | 19,987±3,347 | 14,396±2,178 | 12 |
| MS with CNP | L | O | 15,620±3,538 | 12,599±1,549 | 12 |
| MS with CNP | L | Ta | 14,905±2,509 | 26,693±4,669 | 12 |
| MS with CNP | L | Tm | 13,047±1,674 | 18,803±3,408 | 12 |
| MS with CNP | L | Tp | 12,360±2,317 | 14,068±1,212 | 12 |
| MS with CNP | R | Fp | 21,155±3,095 | 24,039±3,390 | 12 |
| MS with CNP | R | F | 19,081±2,389 | 20,594±2,879 | 12 |
| MS with CNP | R | C | 18,498±2,381 | 17,634±2,711 | 12 |
| MS with CNP | R | P | 21,072±3,297 | 14,396±2,178 | 12 |
| MS with CNP | R | O | 17,894±3,749 | 13,157±1,563 | 12 |
| MS with CNP | R | Ta | 15,841±2,272 | 22,648±2,348 | 12 |
| MS with CNP | R | Tm | 16,592±1,963 | 17,290±2,376 | 12 |
| MS with CNP | R | Tp | 17,588±3,128 | 15,355±4,134 | 12 |
| MS without CNP | L | Fp | 24,736±5,167 | 33,015±5,441 | 12 |
| MS without CNP | L | F | 14,965±1,632 | 22,748±3,938 | 12 |
| MS without CNP | L | C | 14,086±1,202 | 20,192±3,281 | 12 |
| MS without CNP | L | P | 15,654±2,867 | 16,140±3,059 | 12 |
| MS without CNP | L | O | 15,121±2,672 | 17,289±4,231 | 12 |
| MS without CNP | L | Ta | 22,514±4,734 | 33,974±4,730 | 12 |
| MS without CNP | L | Tm | 12,640±2,542 | 23,908±3,632 | 12 |
| MS without CNP | L | Tp | 10,110±1,706 | 19,385±2,781 | 12 |
| MS without CNP | R | Fp | 24,802±3,917 | 32,162±5,050 | 12 |
| MS without CNP | R | F | 15,224±1,483 | 22,261±3,735 | 12 |
| MS without CNP | R | C | 14,595±1,706 | 19,850±3,381 | 12 |
| MS without CNP | R | P | 15,573±2,606 | 15,635±2,792 | 12 |
| MS without CNP | R | O | 14,279±3,237 | 14,783±2,302 | 12 |
| MS without CNP | R | Ta | 23,656±5,209 | 36,333±4,841 | 12 |
| MS without CNP | R | Tm | 12,283±1,541 | 22,384±3,784 | 12 |
| MS without CNP | R | Tp | 13,614±3,102 | 17,286±2,882 | 12 |
| Theta-band | | | | | |
| Healthy Control | L | Fp | 10,137±1,503 | 15,008±1,087 | 12 |
| Healthy Control | L | F | 13,688±2,280 | 16,301±1,225 | 12 |
| Healthy Control | L | C | 11,420±1,692 | 13,534±1,374 | 12 |
| Healthy Control | L | P | 11,356±2,136 | 8,930±1,073 | 12 |
| Healthy Control | L | O | 9,643±1,733 | 8,019±0,763 | 12 |
| Healthy Control | L | Ta | 7,408±1,379 | 14,882±1,205 | 12 |
| Healthy Control | L | Tm | 7,152±1,365 | 13,795±1,251 | 12 |
| Healthy Control | L | Tp | 7,437±1,352 | 11,071±1,331 | 12 |
| Healthy Control | R | Fp | 10,394±1,519 | 14,238±1,023 | 12 |
| Healthy Control | R | F | 13,647±2,238 | 16,770±1,182 | 12 |
| Healthy Control | R | C | 12,367±1,911 | 13,640±1,352 | 12 |
| Healthy Control | R | P | 12,435±2,039 | 8,405±0,848 | 12 |
| Healthy Control | R | O | 11,399±2,512 | 8,069±0,842 | 12 |
| Healthy Control | R | Ta | 7,374±1,228 | 14,639±1,222 | 12 |
| Healthy Control | R | Tm | 8,016±1,538 | 12,855±1,570 | 12 |
| Healthy Control | R | Tp | 9,589±2,295 | 10,494±1,258 | 12 |
| MS with CNP | L | Fp | 29,733±12,377 | 22,495±3,101 | 12 |
| MS with CNP | L | F | 30,298±10,669 | 22,046±2,938 | 12 |
| MS with CNP | L | C | 31,498±10,962 | 17,719±2,483 | 12 |
| MS with CNP | L | P | 31,985±10,595 | 13,412±2,963 | 12 |
| MS with CNP | L | O | 21,760±8,069 | 13,032±2,501 | 12 |
| MS with CNP | L | Ta | 18,057±7,424 | 20,161±3,281 | 12 |
| MS with CNP | L | Tm | 22,316±8,665 | 18,072±2,977 | 12 |
| MS with CNP | L | Tp | 19,401±7,362 | 15,336±3,159 | 12 |
| MS with CNP | R | Fp | 31,570±12,045 | 24,146±2,991 | 12 |
| MS with CNP | R | F | 33,442±11,284 | 23,344±3,202 | 12 |
| MS with CNP | R | C | 34,833±11,537 | 19,393±2,425 | 12 |
| MS with CNP | R | P | 32,104±9,583 | 14,435±2,940 | 12 |
| MS with CNP | R | O | 24,425±7,983 | 13,421±2,535 | 12 |
| MS with CNP | R | Ta | 20,121±6,738 | 21,253±3,220 | 12 |
| MS with CNP | R | Tm | 33,442±11,284 | 18,646±2,834 | 12 |
| MS with CNP | R | Tp | 27,476±8,921 | 14,081±2,616 | 12 |
| MS without CNP | L | Fp | 14,011±3,082 | 17,000±1,990 | 12 |
| MS without CNP | L | F | 15,704±3,497 | 17,945±2,031 | 12 |
| MS without CNP | L | C | 15,880±3,778 | 16,438±2,029 | 12 |
| MS without CNP | L | P | 18,766±5,496 | 13,676±1,951 | 12 |
| MS without CNP | L | O | 14,732±3,887 | 13,229±2,052 | 12 |
| MS without CNP | L | Ta | 9,399±1,671 | 14,389±1,490 | 12 |
| MS without CNP | L | Tm | 9,914±1,955 | 15,526±1,951 | 12 |
| MS without CNP | L | Tp | 9,037±2,244 | 15,058±2,629 | 12 |
| MS without CNP | R | Fp | 15,038±3,557 | 17,682±2,286 | 12 |
| MS without CNP | R | F | 16,459±4,346 | 18,117±2,068 | 12 |
| MS without CNP | R | C | 18,852±6,309 | 16,827±1,969 | 12 |
| MS without CNP | R | P | 21,001±7,359 | 13,583±2,433 | 12 |
| MS without CNP | R | O | 16,026±5,961 | 12,970±2,547 | 12 |
| MS without CNP | R | Ta | 9,426±1,591 | 15,632±1,862 | 12 |
| MS without CNP | R | Tm | 10,656±2,245 | 15,826±1,833 | 12 |
| MS without CNP | R | Tp | 18,975±8,828 | 15,817±3,772 | 12 |
| Alpha-band | | | | | |
| Healthy Control | L | Fp | 24,552±3,515 | 36,244±3,376 | 12 |
| Healthy Control | L | F | 35,221±5,312 | 42,653±2,814 | 12 |
| Healthy Control | L | C | 39,505±4,779 | 48,102±3,036 | 12 |
| Healthy Control | L | P | 87,336±19,803 | 63,772±2,727 | 12 |
| Healthy Control | L | O | 93,801±20,260 | 64,938±3,746 | 12 |
| Healthy Control | L | Ta | 16,661±2,749 | 34,307±3,436 | 12 |
| Healthy Control | L | Tm | 35,221±5,312 | 40,461±3,835 | 12 |
| Healthy Control | L | Tp | 37,460±9,334 | 48,698±4,218 | 12 |
| Healthy Control | R | Fp | 29,494±4,065 | 35,707±3,538 | 12 |
| Healthy Control | R | F | 37,376±4,883 | 43,148±2,564 | 12 |
| Healthy Control | R | C | 44,706±5,504 | 50,756±2,963 | 12 |
| Healthy Control | R | P | 100,209±15,468 | 66,873±2,820 | 12 |
| Healthy Control | R | O | 105,639±21,873 | 68,182±3,142 | 12 |
| Healthy Control | R | Ta | 17,508±2,490 | 36,108±3,512 | 12 |
| Healthy Control | R | Tm | 23,173±3,305 | 39,988±3,358 | 12 |
| Healthy Control | R | Tp | 57,710±16,842 | 56,982±3,284 | 12 |
| MS with CNP | L | Fp | 37,835±10,429 | 32,086±3,952 | 12 |
| MS with CNP | L | F | 46,183±13,666 | 36,120±3,663 | 12 |
| MS with CNP | L | C | 69,386±18,355 | 42,686±4,930 | 12 |
| MS with CNP | L | P | 138,441±42,121 | 50,095±6,913 | 12 |
| MS with CNP | L | O | 94,416±29,482 | 47,547±7,017 | 12 |
| MS with CNP | L | Ta | 25,164±9,744 | 29,980±3,725 | 12 |
| MS with CNP | L | Tm | 41,486±12,061 | 38,895±4,479 | 12 |
| MS with CNP | L | Tp | 44,249±10,231 | 43,180±5,067 | 12 |
| MS with CNP | R | Fp | 41,220±11,200 | 33,264±3,916 | 12 |
| MS with CNP | R | F | 49,697±14,092 | 36,590±3,855 | 12 |
| MS with CNP | R | C | 74,593±19,388 | 43,768±3,985 | 12 |
| MS with CNP | R | P | 132,226±40,941 | 49,231±6,540 | 12 |
| MS with CNP | R | O | 108,086±34,345 | 48,523±6,944 | 12 |
| MS with CNP | R | Ta | 28,541±8,022 | 31,167±3,815 | 12 |
| MS with CNP | R | Tm | 59,578±16,652 | 39,940±4,566 | 12 |
| MS with CNP | R | Tp | 118,406±52,110 | 49,225±5,879 | 12 |
| MS without CNP | L | Fp | 28,905±6,492 | 33,837±4,104 | 12 |
| MS without CNP | L | F | 40,270±8,344 | 42,662±3,875 | 12 |
| MS without CNP | L | C | 52,646±12,612 | 47,185±4,368 | 12 |
| MS without CNP | L | P | 77,186±17,882 | 55,218±4,478 | 12 |
| MS without CNP | L | O | 67,751±19,127 | 52,016±5,443 | 12 |
| MS without CNP | L | Ta | 21,260±3,986 | 31,888±3,582 | 12 |
| MS without CNP | L | Tm | 30,749±7,007 | 42,964±4,157 | 12 |
| MS without CNP | L | Tp | 27,780±4,647 | 46,857±4,452 | 12 |
| MS without CNP | R | Fp | 29,192±5,908 | 33,294±3,879 | 12 |
| MS without CNP | R | F | 39,030±7,700 | 41,876±3,564 | 12 |
| MS without CNP | R | C | 55,064±14,462 | 46,810±4,176 | 12 |
| MS without CNP | R | P | 83,124±22,428 | 55,824±4,367 | 12 |
| MS without CNP | R | O | 72,521±21,562 | 55,280±4,264 | 12 |
| MS without CNP | R | Ta | 19,916±3,642 | 30,374±2,926 | 12 |
| MS without CNP | R | Tm | 33,254±7,340 | 43,966±3,984 | 12 |
| MS without CNP | R | Tp | 63,945±21,319 | 50,649±5,222 | 12 |
| Beta1-band | | | | | |
| Healthy Control | L | Fp | 4,776±0,603 | 8,022±1,011 | 12 |
| Healthy Control | L | F | 6,558±0,954 | 8,536±0,788 | 12 |
| Healthy Control | L | C | 7,776±1,162 | 9,239±0,589 | 12 |
| Healthy Control | L | P | 11,358±2,363 | 8,565±0,794 | 12 |
| Healthy Control | L | O | 8,911±1,949 | 7,723±0,885 | 12 |
| Healthy Control | L | Ta | 3,911±0,571 | 9,061±1,092 | 12 |
| Healthy Control | L | Tm | 5,097±0,726 | 11,110±0,962 | 12 |
| Healthy Control | L | Tp | 6,737±1,148 | 10,900±1,347 | 12 |
| Healthy Control | R | Fp | 5,042±0,698 | 7,803±1,083 | 12 |
| Healthy Control | R | F | 6,776±1,003 | 8,815±0,689 | 12 |
| Healthy Control | R | C | 8,381±1,528 | 9,120±0,621 | 12 |
| Healthy Control | R | P | 10,921±1,840 | 7,497±0,724 | 12 |
| Healthy Control | R | O | 9,488±2,048 | 7,558±1,059 | 12 |
| Healthy Control | R | Ta | 3,995±0,509 | 9,184±1,051 | 12 |
| Healthy Control | R | Tm | 6,031±1,046 | 10,531±1,310 | 12 |
| Healthy Control | R | Tp | 7,364±1,634 | 8,496±0,657 | 12 |
| MS with CNP | L | Fp | 10,197±2,360 | 9,687±1,263 | 12 |
| MS with CNP | L | F | 11,029±2,078 | 10,565±1,286 | 12 |
| MS with CNP | L | C | 13,171±2,388 | 10,094±1,393 | 12 |
| MS with CNP | L | P | 18,413±4,697 | 9,951±1,830 | 12 |
| MS with CNP | L | O | 12,687±2,884 | 9,777±1,300 | 12 |
| MS with CNP | L | Ta | 6,873±1,826 | 10,097±1,226 | 12 |
| MS with CNP | L | Tm | 9,148±1,852 | 10,714±1,361 | 12 |
| MS with CNP | L | Tp | 9,203±1,376 | 11,097±1,379 | 12 |
| MS with CNP | R | Fp | 10,534±2,111 | 9,963±1,180 | 12 |
| MS with CNP | R | F | 11,820±2,134 | 10,527±1,180 | 12 |
| MS with CNP | R | C | 14,071±2,428 | 10,836±1,187 | 12 |
| MS with CNP | R | P | 17,140±3,480 | 9,622±1,311 | 12 |
| MS with CNP | R | O | 14,696±2,927 | 10,361±1,802 | 12 |
| MS with CNP | R | Ta | 9,201±1,868 | 11,733±1,066 | 12 |
| MS with CNP | R | Tm | 12,802±2,431 | 10,974±1,229 | 12 |
| MS with CNP | R | Tp | 16,481±3,975 | 12,285±2,483 | 12 |
| MS without CNP | L | Fp | 6,586±1,134 | 8,539±1,035 | 12 |
| MS without CNP | L | F | 7,960±1,272 | 9,840±1,087 | 12 |
| MS without CNP | L | C | 8,655±1,460 | 9,753±1,163 | 12 |
| MS without CNP | L | P | 9,284±1,275 | 9,205±1,576 | 12 |
| MS without CNP | L | O | 8,724±1,384 | 9,176±1,635 | 12 |
| MS without CNP | L | Ta | 5,736±0,987 | 9,186±1,355 | 12 |
| MS without CNP | L | Tm | 7,960±1,272 | 10,113±1,136 | 12 |
| MS without CNP | L | Tp | 5,775±1,132 | 10,230±1,278 | 12 |
| MS without CNP | R | Fp | 7,423±1,264 | 8,978±1,102 | 12 |
| MS without CNP | R | F | 8,401±1,402 | 10,237±1,098 | 12 |
| MS without CNP | R | C | 8,885±1,420 | 10,058±1,149 | 12 |
| MS without CNP | R | P | 9,804±1,562 | 9,160±1,462 | 12 |
| MS without CNP | R | O | 9,135±1,856 | 9,399±1,602 | 12 |
| MS without CNP | R | Ta | 5,768±0,983 | 9,558±1,111 | 12 |
| MS without CNP | R | Tm | 6,847±1,097 | 10,791±1,106 | 12 |
| MS without CNP | R | Tp | 7,572±1,477 | 9,410±1,450 | 12 |
| Beta2-band | | | | | |
| Healthy Control | L | Fp | 4,044±0,717 | 6,941±1,215 | 12 |
| Healthy Control | L | F | 5,067±0,728 | 7,180±1,117 | 12 |
| Healthy Control | L | C | 5,723±0,876 | 7,370±0,965 | 12 |
| Healthy Control | L | P | 5,714±1,168 | 4,748±0,551 | 12 |
| Healthy Control | L | O | 4,958±0,854 | 5,348±1,098 | 12 |
| Healthy Control | L | Ta | 4,370±1,549 | 9,067±1,796 | 12 |
| Healthy Control | L | Tm | 4,199±0,950 | 8,878±1,273 | 12 |
| Healthy Control | L | Tp | 4,637±1,116 | 8,831±2,501 | 12 |
| Healthy Control | R | Fp | 4,139±0,574 | 6,661±1,154 | 12 |
| Healthy Control | R | F | 4,532±0,537 | 6,789±1,131 | 12 |
| Healthy Control | R | C | 4,891±0,569 | 6,202±0,830 | 12 |
| Healthy Control | R | P | 5,344±0,694 | 4,191±0,637 | 12 |
| Healthy Control | R | O | 4,851±0,834 | 5,016±1,305 | 12 |
| Healthy Control | R | Ta | 3,335±0,641 | 8,056±1,547 | 12 |
| Healthy Control | R | Tm | 4,250±0,745 | 8,521±1,632 | 12 |
| Healthy Control | R | Tp | 4,109±0,731 | 6,266±1,317 | 12 |
| MS with CNP | L | Fp | 8,097±1,574 | 10,945±2,391 | 12 |
| MS with CNP | L | F | 9,069±2,451 | 11,544±3,817 | 12 |
| MS with CNP | L | C | 12,955±4,455 | 13,468±5,512 | 12 |
| MS with CNP | L | P | 14,838±3,823 | 14,295±5,524 | 12 |
| MS with CNP | L | O | 13,749±3,674 | 17,045±6,139 | 12 |
| MS with CNP | L | Ta | 7,020±1,539 | 13,069±2,881 | 12 |
| MS with CNP | L | Tm | 8,159±1,716 | 13,515±4,181 | 12 |
| MS with CNP | L | Tp | 10,053±2,158 | 16,319±4,657 | 12 |
| MS with CNP | R | Fp | 6,823±1,037 | 8,587±1,793 | 12 |
| MS with CNP | R | F | 7,250±1,016 | 8,946±2,288 | 12 |
| MS with CNP | R | C | 4,455±7,440 | 8,368±2,201 | 12 |
| MS with CNP | R | P | 11,216±1,812 | 12,316±4,870 | 12 |
| MS with CNP | R | O | 11,161±1,615 | 14,539±4,995 | 12 |
| MS with CNP | R | Ta | 7,570±1,453 | 13,199±3,868 | 12 |
| MS with CNP | R | Tm | 10,247±2,429 | 13,150±4,644 | 12 |
| MS with CNP | R | Tp | 8,647±1,530 | 9,055±2,617 | 12 |
| MS without CNP | L | Fp | 6,175±2,077 | 7,609±1,555 | 12 |
| MS without CNP | L | F | 5,614±1,521 | 6,805±1,174 | 12 |
| MS without CNP | L | C | 5,586±1,250 | 6,432±1,027 | 12 |
| MS without CNP | L | P | 5,538±0,859 | 5,761±1,029 | 12 |
| MS without CNP | L | O | 7,931±1,980 | 8,290±1,823 | 12 |
| MS without CNP | L | Ta | 6,822±1,769 | 10,562±2,415 | 12 |
| MS without CNP | L | Tm | 4,377±0,863 | 7,489±1,071 | 12 |
| MS without CNP | L | Tp | 4,651±1,051 | 8,469±1,301 | 12 |
| MS without CNP | R | Fp | 6,695±2,131 | 7,883±1,852 | 12 |
| MS without CNP | R | F | 6,314±1,873 | 7,508±1,426 | 12 |
| MS without CNP | R | C | 5,404±1,124 | 6,456±1,115 | 12 |
| MS without CNP | R | P | 5,731±1,219 | 5,798±1,264 | 12 |
| MS without CNP | R | O | 7,400±2,610 | 7,567±2,057 | 12 |
| MS without CNP | R | Ta | 4,639±0,985 | 8,103±1,655 | 12 |
| MS without CNP | R | Tm | 4,431±0,991 | 7,034±1,058 | 12 |
| MS without CNP | R | Tp | 5,273±1,191 | 6,837±1,114 | 12 |
